# Effects of duck fat and κ-carrageenan as replacements for beef fat and pork backfat in frankfurters

**DOI:** 10.5713/ab.21.0378

**Published:** 2022-01-04

**Authors:** Dong-Min Shin, Jong Hyeok Yune, Yea Ji Kim, Sang Hoon Keum, Hyun Su Jung, Hyuk Cheol Kwon, Do Hyun Kim, Hyejin Sohn, Chang Hee Jeong, Hong Gu Lee, Sung Gu Han

**Affiliations:** 1 Department of Food Science and Biotechnology of Animal Resources, Konkuk University, Seoul 05029, Korea; 2 Department of Animal Science and Technology, Konkuk University, Seoul 05029, Korea; 3 Microbiology and Functionality Research Group, World Institute of Kimchi, Gwangju 61755, Korea

**Keywords:** Duck Fat, κ-Carrageenan, Rheological Property, Fat Replacement

## Abstract

**Objective:**

Frankfurters are emulsion-type sausages that are widely consumed worldwide. However, some concerns regarding negative health effects have been raised because of the high fat content and the type of fat. This study aimed to evaluate the effects of duck fat and κ-carrageenan as replacements for beef fat and pork backfat in frankfurters.

**Methods:**

The different formulations for the frankfurters were as follows: 20% beef fat (BF), 20% pork backfat (PBF), 20% duck fat (DF), 20% soybean oil (SO), 20% duck fat/1% κ-carrageenan (DFC), and 20% soybean oil/1% κ-carrageenan (SOC). Physicochemical (fatty acid profile, color, rheological properties, cooking loss, water holding capacity, emulsion stability, and texture profile analysis), oxidative stability and sensory properties of frankfurters were evaluated.

**Results:**

Duck fat and κ-carrageenan improved rheological properties of meat batter, and physicochemical properties (emulsion stability, cooking loss, and hardness) of frankfurters. Moreover, duck fat added-frankfurters (DF and DFC) had higher oxidative stability than that of soybean-added frankfurters (SO and SOC) during refrigerated storage for 28 days. In sensory evaluation, flavor, texture, and overall acceptability of DFC were acceptable to untrained panelists.

**Conclusion:**

Our data suggest that duck fat and κ-carrageenan can replace beef fat and pork backfat in frankfurters. Duck fat and κ-carrageenan contributed to improve the physicochemical properties and oxidative stability while maintaining sensory properties. Therefore, the use of duck fat and κ-carrageenan may be a suitable alternative for replacing beef fat or pork backfat in frankfurters.

## INTRODUCTION

Frankfurters are emulsified processed meat products that are widely consumed in several countries because of their flavor, convenience, and inexpensive pricing [1]. During their manufacture, animal fats (e.g., beef fat and pork backfat) are key ingredients that contribute to improve the textural and sensory properties. However, excessive animal fat intake has been associated with various lifestyle diseases—such as cardiovascular disease, type-2 diabetes, and cancer—due to the high content of saturated fatty acids in animal fats [2]. Consequently, the World Health Organization recommended to increase the consumption of unsaturated fat and limit the intake of saturated fat, resulting in growing consumer interest in alternative meat products [3]. This motivated researchers in recent years to develop low-fat meat products or meat products containing more unsaturated fatty acids.

Although duck fat is an animal fat, it is a good source of unsaturated fat due to its higher levels of unsaturated fatty acids (64.51%) as well as lower levels of saturated fatty acids (28.53%) than beef fat and pork backfat, which contain 38.75% and 36.40% saturated fatty acids, respectively [4]. Moreover, duck fat has higher levels of oleic acid (48.7%) and lower levels of linoleic acid (15.08%) than conventional vegetable oils [4]. Thus, intake of duck fat may provide considerable benefits to human health, such as diminishing the risk of cardiovascular diseases because of the oleic acid present in duck fat [5]. Another benefit of using duck fat in meat products is its prolonged shelf-life and physicochemical stability. Unlike the linoleic acid present in vegetable oils, oleic acid is more resistant to oxidation and rancidity [6].

Processed meats containing vegetable oil that can nega tively affect textural and sensory quality properties because of its low hardness and sticky texture [7,8]. To overcome these limitations, various food additives have been employed; for example, gums, protein, starch, and fiber [9]. In particular, the addition of hydrocolloid gums (e.g., carrageenan, konjac, xanthan gum, locust bean gum, gellan gum, flaxseed gum, and curdlan) was the most efficacious, economical, and convenient technique for enhancing the textural or gelling properties of emulsified meat products [10]. According to a previous study, the addition of hydrocolloids improved the textural properties of emulsified meat products [11]. κ-Carrageenan is a general anionic polysaccharide that is commonly extracted from tropical red seaweeds and contains 22% (w/w) ester sulfate [12]. In addition, κ-carrageenan is one of the most widely used additives in the meat industry because of its exceptional water holding, thickening, and gelling capacities [13]. It was also demonstrated that κ-carrageenan improved the sensory properties by enhancing textural properties of emulsified processed meat [10].

Considering the positive health effects of duck fat and the outstanding properties of κ-carrageenan as an additive in emulsified meat products, the combined use of duck fat and κ-carrageenan might be an appropriate strategy for the development of healthy meat products. Therefore, this study aimed to investigate the effects of duck fat and κ-carrageenan on the physicochemical characteristics and oxidative stability of frankfurters.

## MATERIALS AND METHODS

### Preparation of frankfurters

Duck fat was provided by Taekyung Nongsan Co., Ltd. (Seoul, Korea). Beef fat, pork backfat, fresh pork ham, and soybean oil were purchased from a local market. The duck fat (20%) as well as beef fat, pork backfat, and soybean oil were used to prepare meat batters and frankfurters, in order to compare the effects of the varying levels of saturated and unsaturated fatty acids on the quality characteristics of these meat products. κ-Carrageenan was also added to frankfurters to improve their physicochemical properties. Supplementation with 1% κ-carrageenan is known to be optimal for improving the physicochemical properties of emulsified meat products [14]. Consequently, six different formulations of frankfurters were prepared: 20% beef fat (BF), 20% pork backfat (PBF), 20% duck fat (DF), 20% soybean oil (SO), 20% duck fat/1% κ-carrageenan (DFC), and 20% soybean oil/1% κ-carrageenan (SOC). The other ingredients used were as follows: lean meat (60%), ice (20%), salt (1.5%), nitrite (0.015%), ascorbic acid (0.05%), tripolyphosphate (0.3%), and spices (0.8%). All visible connective tissue, subcutaneous fat, and intramuscular fat were trimmed from the meat. To process the frankfurters, lean meat, beef fat and pork backfat were minced through a 3-mm plate using a mincer machine (PM-70; Mainca, Barcelona, Spain). The minced meat along with the fats, soybean oil, ice, and other ingredients were emulsified using a silent cutter (Cutter C4 VV; SIRMAN, Venezia, Italy). The frankfurter batter was stuffed in a 240-mm collagen casing (NIPPI Inc., Tokyo, Japan) using a stuffer (IS-8; Sirman, Marsango, Italy). They were subsequently cooked in a smokehouse at 80°C for 30 min, until the core temperature reached 72°C, followed by cooling to room temperature (25°C±1°C) for 3 h. Finally, the frankfurters were vacuum-packed in polyethylene bags and stored at 4°C±1°C for 28 d. In each group, 1.5 kg of emulsified meat batter was manufactured and obtained 15 to 16 frankfurters.

### Fatty acid profile

The fatty acid profile analysis of each frankfurter and raw fat/oil sample was performed. Briefly, fat and oil were extracted using chloroform and methanol solvents (2:1, v/v). After fat and oil extraction, boron trifluoride/methanol solution was used for methyl esterification of the samples. The fatty acid profile was analyzed using a gas chromatograph/FID (HP6890 series GC System; Agilent technologies, CA, USA) equipped with a Sp-2560 capillary column (100 m× 0.25 mm×0.2 μm, film thickness), and FAME Mix STANDARD (Sigma-Aldrich/47885-U, St. Louis, Mo, USA) was used as standard for measuring the fatty acid contents and ratios. Helium was used as the carrier gas at a flow rate of 1.2 mL/min (injection volume, 1.0 μL). The injector and detector (FID System) temperatures were programmed at 255°C and 260°C, respectively. Furthermore, the oven temperature was programmed at 70°C to 100°C: 5/min (hold: 2 min), 100°C to 175°C: 10/min (hold 40 min), and 175°C to 225°C: 5/min (hold: 40 min).

### pH and color measurements

A total of 5 g of the samples was mixed in 20 mL distilled water and homogenized at 10,000 rpm for 1 min using a homogenizer (DAIHAN Scientific Co., Ltd., Gangwon, Korea). Subsequently, the pH of the supernatants was determined using a LAQUA pH meter (Horiba, Ltd., Kyoto, Japan).

The color measurement was conducted on the surface of frankfurter samples using a CR-210 colorimeter (Minolta Camera Co., Ltd., Osaka, Japan) during the storage period. The samples were bloomed for 30 min at RT, and the color was expressed as L* (lightness), a* (redness), and b* (yellowness) values.

### Rheological properties

The viscoelasticity and apparent viscosity of the frankfurter batter were measured using an MCR 92 rheometer (Anton Paar, Graz, Austria) (n = 3/group). The storage modulus (G′) and loss modulus (G″) were determined in the frequency range of 0.1 to 100 Hz using a PP25 parallel plate (25-mm diameter) at 25°C. The results were expressed in Pascal (Pa) units. Furthermore, the apparent viscosity of the batters was determined at 25°C (n = 3/group), and data were collected between shear rates of 0.1 to 100 1/s. These results were expressed in Pascal-seconds (Pa·s). The data were finally analyzed using the Anton Paar RheoCompass Ver. 1.25 software.

### Emulsion stability and water holding capacity of meat batters and cooking loss of frankfurters

For measuring emulsion stability of the meat batter, graduated glass tubes were placed in the middle of a 15-mesh sieve (4 cm×4 cm) and filled with meat batter. The tubes were subsequently sealed and heated in a water bath at 80°C for 30 min. After cooling, the graduation of water fluid and separated fat layer in the tubes was calculated in order to determine the separation of water and lipids from the meat batter. The emulsion stability was calculated using the following equation:


$$\begin{array}{l} \text{Water released }(\%)=[\text{separated water fluid }(\text{mL})/\text{weight of raw meat batter }(\text{g})]\times 100\\ \text{Fat released }(\%)=[\text{separated fat layer }(\text{mL})/\text{weight of raw meat batter }(\text{g})]\times 100\\ \text{Emulsion stability}=\text{water released}+\text{fat released} \end{array}$$

For determining water holding capacity (WHC) of the samples, 10 g of meat batter was placed into a conical tube and centrifuged at 6,000×g for 15 min at 4°C. Subsequently, the tube was heated in a water bath at 85°C for 15 min and cooled to room temperature. After cooling, the samples were centrifuged at 6,000×g for 15 min at 4°C, following which the samples were removed and weighed. WHC was calculated using the following equation:


WHC (%)=[(M2-M)/M1-M]×100

M = Weight of the empty tube (g)
M1 = total weight of the meat batter + tube (g)
M2 = total weight of the meat batter + tube after heating and centrifugation (g)

To determine cooking loss, frankfurter sample s were heated at 80°C for 30 min. The cooked samples were subsequently allowed to cool to room temperature (25°C±1°C) for 2 h. After cooling, cooking loss was measured using the following equation:


Cooking loss (%)=[(weight of raw meat batter [g]-weight of cooked meat batter [g])/weight of raw meat batter (g)]×100

### Texture profile analysis

Texture profile analysis (TPA) was conducted using a TA-XT2i texture analyzer (Stable Micro Systems Ltd., Surrey, England) equipped with a 45° conical probe. The frankfurter samples were cut into 2.5×2.0 cm (diameter×height)-pieces (n = 6/group). Thereafter, the TPA was analyzed according to the following program parameters: pre-test speed 2.0 mm/s, test speed 2.0 mm/s, post-test speed 8.0 mm/s, maximum load 2 kg, distance 20 mm, and force 5 g. The results were analyzed using maximum-recorded force (g).

### Thiobarbituric acid reactive substance value

The frankfurter sample (10 g) was mixed with distilled water (50 mL) and then homogenized for 2 min using a Model AM-7 homogenizer (Nissei Co., Ltd., Tokyo, Japan). The mixture was subsequently transferred to a distillation tube and distilled water (47.5 mL) was added, along with 4 N HCl aqueous solution (2.5 mL) and an antifoam agent (1 mL, KMK-73; Shin-Etsu Silicone Co., Ltd., Seoul, Korea). The samples were distilled, and 40 mL of distillate was collected. The collected sample (5 mL) was mixed with thiobarbituric acid (TBA) reagent (5 mL, 0.02 M in 90% acetic acid) reagent in a test tube and heated in a water bath at 95°C for 30 min. After cooling, the absorbance of the samples was measured at 538 nm using a UV/VIS spectrophotometer (Optizen 2120 UV Plus; Mecasys Co., Ltd., Daejeon, Korea).

### Sensory evaluation

The sensory evaluation of frankfurters was performed by 17 untrained panelists (9 males and 8 females; ages from 24 to 32; students at Konkuk University Food Science Department). The samples were identified using a 3-digit random code and served to the panelists. Consequently, they were evaluated for their appearance, flavor, aroma, juiciness, and overall acceptability using a 9-point hedonic scale (1 = extremely dislike and 9 = extremely like).

### Statistical analyses

Data are expressed as the mean±standard deviation. Statistical significance of all the data was analyzed using one-way analysis of variance with the SPSS Ver. 24.0 software (SPSS Inc., Chicago, IL, USA), except thiobarbituric acid reactive substance (TBARS) data which were analyzed using two-way analysis of variance. The Duncan’s multiple range test was conducted to determine significant differences (p<0.05) between the groups.

## RESULTS AND DISCUSSION

### Fatty acid profile of frankfurters and raw fat/oil

In recent decades, there has been an increasing demand for healthier diets. To this end, reducing animal fats and providing better fatty acid profiles in the diet are both practical strategies for the development of healthy meat products. The fatty acid profiles of frankfurters and raw fat/oil presented in Table 1. In comparison to beef fat and pork backfat, duck fat was observed to contain increased amounts of unsaturated fatty acids (67.19%) and the lowest amount of saturated fatty acids (32.81%) (p<0.05). Similarly, the fat profile of duck fat appears to be better than that of other animal fats (e.g., bovine fat and swine fat). For instance, in a previous study, duck fat extracted from duck skin contained higher unsaturated fatty acids and lower saturated fatty acids than in the bovine fat and swine fat extracted from bovine skin and swine skin, respectively [4]. In the frankfurter groups of the present study, there were significant differences in the fatty acid profile depending on the fat content (p<0.05). Duck fat-added frankfurters showed lower levels of saturated fatty acids (33.28% in DF and 33.26% DFC) than beef fat or pork backfat-added frankfurters (36.58% in BF and 37.34% in PBF; p<0.05). Duck fat-added frankfurters exhibited higher unsaturated fatty acid content (66.72% in DF and 66.74% in DFC) than beef fat or pork backfat-added frankfurters (63.42% in BF and 62.66% in PBF). In detail, duck fat-added frankfurters exhibited higher total monounsaturated fatty acid contents (48.85% in DF and 48.96% in DFC) and lower polyunsaturated fatty acid contents (17.85% in DF and 17.7% DFC) than those of SBO and SOC. Moreover, the addition of κ-carrageenan did not influence the fatty acid profiles of frankfurters. Soybean oil had the highest unsaturated fatty acid (83.85%) and polyunsaturated fatty acid contents (70.86%) compared with other fats. Furthermore, SO and SOC samples contained the highest amounts of unsaturated fatty acids (82.28% in SO and 81.40% in SOC; p<0.05). The most commonly occurring unsaturated fatty acid was linoleic acid in SO and SOC, whereas oleic acid was the highest unsaturated fatty acid in animal fat-added frankfurters (i.e., BF, PBF, DF, and DFC). Overall, the fatty acid profile of duck fat was desirable in frankfurters because of higher levels of unsaturated fatty acids (e.g., oleic acid and linoleic acid) and lower levels of saturated fatty acids (e.g., stearic acid) than in beef fat or pork backfat-added frankfurters.

### pH and color of frankfurters

The pH of the frankfurters is listed in Table 2. The pH of all frankfurters was identified to be 6.06 to 6.07, and this pH value was considered acceptable in cooked frankfurters [15]. Because the pH was not significantly different among the groups (p>0.05), we demonstrated that the fats/oil and κ-carrageenan used in this study minimally affected the pH of the frankfurters.

The color of frankfurters is an important factor in evalu ating the quality of meat products and immensely influences consumer choices. The color parameters of the frankfurters are listed in Table 2. Duck fat and soybean oil-added frankfurters (DF, DFC, SO, and SOC) had relatively lighter coloring among all the groups (p<0.05). This result might be explained by the differences in the droplet size of each fat or oil employed in the preparation of these frankfurters. The increased lightness in the color of meat products is related to greater light reflection, attributed to oil-based emulsions normally possessing smaller droplet diameters than animal fat-based emulsions [16]. In fact, the droplet size of duck fat is larger than that of vegetable oil but smaller than that of beef fat and pork backfat [17]. The addition of κ-carrageenan increased the lightness of frankfurters (e.g., DF vs DFC), in accordance with existing data on the light scattering effect of polysaccharides (e.g., carrageenan and konjac) capable of heightening the lightness of emulsified meat products [10,18]. In addition, a previous study on κ-carrageenan-added sausages indicated higher lightness values in low-fat and low-sodium sausages [19]. Furthermore, the redness and yellowness values of DF and DFC frankfurters were, respectively, lower, and higher than that of BF and PBF. The changes in redness of frankfurters can be explained by the oxidation during the cooking process. Lipid oxidation could influence the oxidation of myoglobin, leading to decrease in redness of meat [20]. In the current study, high oxidative stability of oleic acid in duck fat may lead to decrease of myoglobin oxidation during cooking processing. κ-Carrageenan-added frankfurter groups (DFC and SOC) had the highest yellowness compared with other groups. Similar result reported that the fermented sausage with κ-carrageenan had higher yellowness than those without κ-carrageenan due to its intrinsic color [21].

### Rheological properties of frankfurter batters

**Figure 1:**
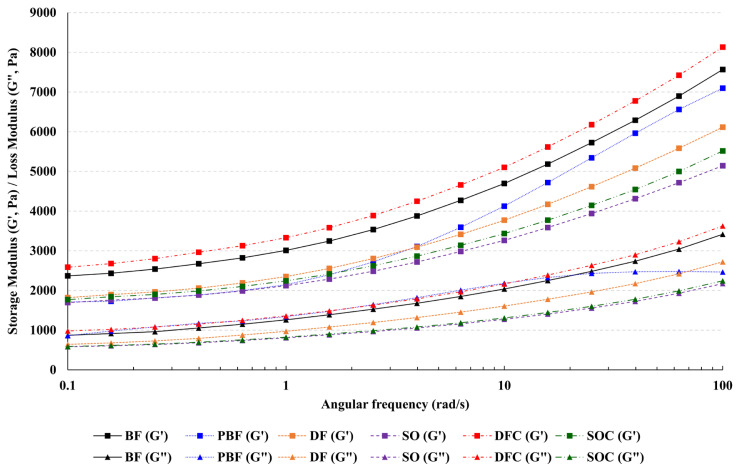
Viscoelasticity of the frankfurter batters. The storage modulus (G′) and loss modulus (G″) of meat batters were determined in the frequency range of 0.1 to 100 Hz at 25°C (n = 3). BF (20% beef fat), PBF (20% pork back fat), DF (20% duck fat), SO (20% soybean oil), DFC (20% duck fat/1% κ-carrageenan), and SOC (20% soybean oil/1% κ-carrageenan).

The viscoelasticity of the frankfurter batters is depicted in Figure 1. In solid-like rheological properties, the G′ (storage modulus) is higher than G″ (loss modulus), whereas G″ is higher than G′ inliquid-like rheological properties [22]. In our data, the G′ value was higher than G″ in all the samples, indicating that they possessed a strong gel-like property. Both moduli exhibited a direct correlation with the frequency; that is, they increased proportionately with the frequency. Moreover, κ-carrageenan further facilitated the increase in G′ and G″ values of frankfurter batters. DFC displayed the highest G′ and G″ values at all frequencies compared to the other samples (p<0.05), whereas SO and SOC exhibited relatively lower G′ and G″ values than DF and DFC (p<0.05). These results may be attributed to the differences in the melting points of duck fat and soybean oil. According to a previous study, meat emulsions prepared with duck fat demonstrated higher G′ and G″ values than those prepared with vegetable oil because of its higher melting point [17]. κ-Carrageenan is a carbohydrate that can provide higher elasticity in emulsified meat products, and hence, it should be capable of increasing the viscoelasticity of frankfurters as well [10].

**Figure 2:**
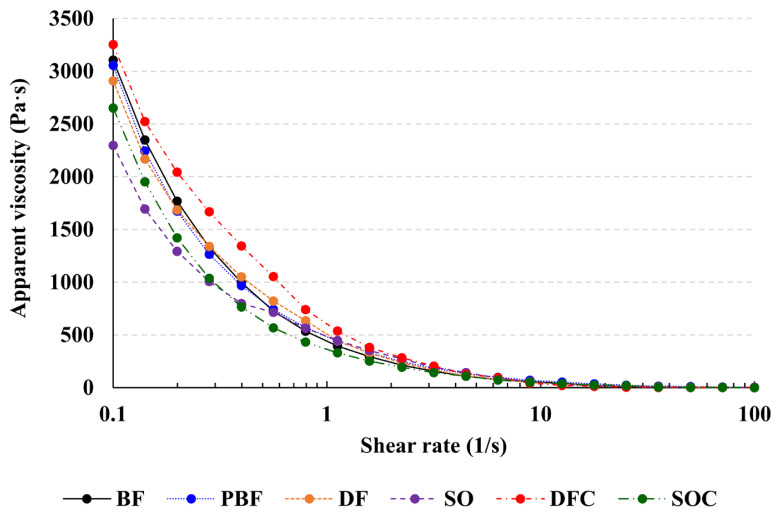
Apparent viscosity of the frankfurter batters. The apparent viscosity of meat batters was determined at 25°C (n = 3). BF (20% beef fat), PBF (20% pork back fat), DF (20% duck fat), SO (20% soybean oil), DFC (20% duck fat/1% κ-carrageenan), and SOC (20% soybean oil/1% κ-carrageenan).

The apparent viscosity of the frankfurter batter samples shared similarities with the viscoelasticity data (Figure 2). In particular, the apparent viscosity of DFC was higher than that of the other frankfurter groups (p<0.05). The apparent viscosity of meat emulsions is influenced by fat type and fat concentration [23]. The increase in the apparent viscosity of meat products is also correlated with strong emulsion stability [24]. Therefore, it can be deduced that the addition of κ-carrageenan increased the viscosity of emulsified meat batters. In fact, the meat batter supplemented with hydrocolloid had a higher viscosity because of the improved binding capacity between protein and water [11]. In summary, our data indicate that the incorporation of duck fat and κ-carrageenan can enhance the rheological properties of frankfurters.

### Emulsion stability and water holding capacity of meat batters and cooking loss of frankfurters

Table 3 depicts the emulsion stability and WHC of the frankfurter batters. The emulsion stability of frankfurter batters was evaluated by measuring water, fat, and total released fluid. Emulsion stability was markedly enhanced in DF compared to BF, PBF, and SO. The addition of κ-carrageenan further improved the emulsion stability (DF vs DFC and SO vs SOC; p<0.05). Moreover, water and total released fluid were significantly lower in the DF group than in the other groups, whereas fat release did not differ significantly among the groups (p>0.05). Among the various batters evaluated, DFC and SOC exhibited the highest emulsion stability (p<0.05). The increased emulsion stability of frankfurters may be attributed to the role of κ-carrageenan in holding water and fat in the batter matrix during heating [10]. In particular, κ-carrageenan may induce the synthesis of hydrocolloids and increase the viscosity of the continuous phase surrounding fat droplets in emulsified meat batter [25]. Consequently, the emulsion can interfere with the movement of water or fat droplets. The emulsion stability of DF was observed to be higher than that of SO (p<0.05). This result can be ascribed to the heat stability of duck fat, which has a higher melting point than vegetable oil [17].

WHC can affect the physical properties of frankfurters [1]. As depicted in Table 3, the type of fats/oil also significantly affected the WHC of frankfurter batters. Higher WHC was observed in DF and SO than in BF and PBF (p<0.05). In addition, WHC increased with the addition of κ-carrageenan to frankfurter meat batters (i.e., DF vs DFC and SO vs SOC) (p<0.05). A previous study has established that κ-carrageenan can improve the WHC of meat products [26]. Moreover, soybean protein-myofibrillar protein gel demonstrated nearly 100% WHC when 1% κ-carrageenan was added [27]. This elevation in the WHC may be linked to the high emulsion stability and low cooking loss observed in DFC and SOC. The WHC of emulsified meat products can contribute to cooking loss and emulsion stability of products during the heating process [28].

Cooking loss is an important factor to be considered with regard to emulsified meat products. It measures the ability of emulsified meat products to retain their juices during cooking and can affect the textural properties of emulsified meat products [23]. The type of fat or oil present in frankfurters are known to affect this cooking loss. As presented in Table 3, DFC had the lowest cooking loss compared with other groups (p<0.05). This may be attributed to the differences in the droplet sizes of the fat or oil emulsions. In this respect, the fat emulsion was more stable to thermal or physical shock than the oil emulsion because larger fat globules were fixed to the fat phase post-heating or physical shock [17,29]. Furthermore, it was observed that the addition of κ-carrageenan significantly decreased the cooking loss of frankfurters (p< 0.05). To this end, the DFC samples exhibited the lowest cooking loss (p<0.05) because of the high water retention capacity of κ-carrageenan. According to a previous study, κ-carrageenan minimized the cooking loss of beef gels as more water was retained in a stronger gel network [30]. Consequently, it can be concluded that the use of duck fat and κ-carrageenan in frankfurters could replace beef fat or pork backfat without damaging emulsion stability, WHC, and cooking loss of frankfurters.

### Texture profile analysis of frankfurters

The TPA was performed on the frankfurters, whereby the hardness, springiness, chewiness, and gumminess of the frankfurters were found to be significantly affected (p<0.05; Table 4). DF exhibited relatively lower levels of the above properties than those in BF or PBF. However, DFC had higher hardness, chewiness, and gumminess than the other groups (p<0.05). SO demonstrated the lowest TPA parameters compared to the other groups because of the lower melting point and higher level of unsaturated fatty acids in SO (p<0.05). In general, fats play a critical role in the texture of emulsified meat products because of their ability to provide stability to emulsified meat products [23,31]. In particular, the type and level of fat are important for emulsion stability and texture. Moreover, the textural properties of emulsified meat products have been attributed to cooking loss, WHC, and rheological properties [23,32]. The data obtained in the present study indicated that the addition of κ-carrageenan to DF positively affected the textural properties of frankfurters. It has been suggested that the polymerization of κ-carrageenan and its interactions with myofibrillar proteins are primarily responsible for the promotion of TPA parameters during the heat-induced gelation process [33]. Therefore, our data proposed that the addition of duck fat and κ-carrageenan can effectively replace beef fat or pork backfat in frankfurters with maintaining their textural properties.

### Thiobarbituric acid reactive substance value of frankfurters

**Figure 3:**
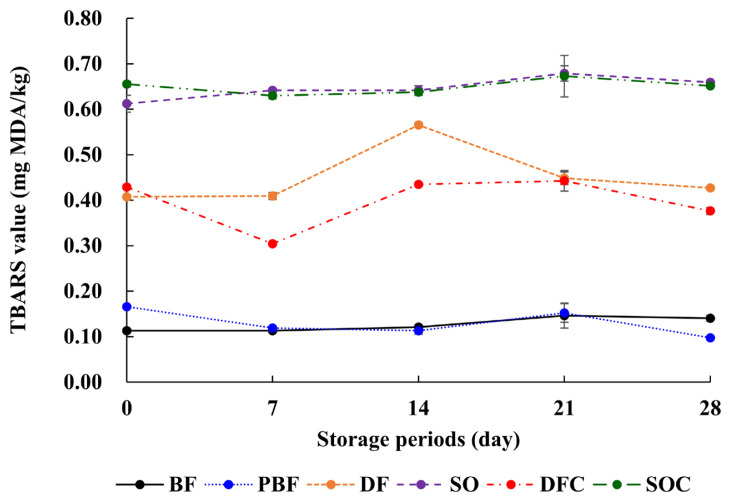
TBARS values of frankfurters during storage at 4°C±1°C for up to 28 d. The error bars indicate SD (n = 3). BF (20% beef fat), PBF (20% pork back fat), DF (20% duck fat), SO (20% soybean oil), DFC (20% duck fat/1% κ-carrageenan), and SOC (20% soybean oil/1% κ-carrageenan).

Lipid oxidation is closely associated with rancidity, off-putting flavor, and discoloration in meat products. The TBARS value is an indicator for evaluating the degree of secondary lipid oxidation products, such as malondialdehyde (MDA). TBARS values were assessed in frankfurters while in cold storage for 28 d. The variations in the TBARS values of frankfurters are depicted in Figure 3. Both SO and SOC showed the highest TBARS values during storage (p<0.05), whereas TBARS values were lower in DF and DFC than in SO and SOC. Moreover, κ-carrageenan did not affect the TBARS value. The lowest TBARS value was observed in the case of frankfurters supplemented with BF and PBF (p<0.05). All frankfurter samples exhibited TBARS values below 1.0 mg MDA/kg sample, at which initial rancidity can occur [34]. Furthermore, it has been reported that TBARS values are generally lower in higher saturated fatty acids (e.g., beef fat or pork backfat) than unsaturated fatty acids (e.g., duck fat and chicken fat) [4]. Moreover, the type of unsaturated fatty acid could affect the variations in TBARS values. The DF and DFC groups contained higher levels of saturated fatty acids and oleic acids than the SO and SOC groups, resulting in lower TBARS values in DF and DFC than in the latter groups. In particular, linoleic acid is about ten-times more vulnerable to lipid oxidation than oleic acid [6]. Concurrently, the data obtained in this study demonstrated that meat products containing high levels of oleic acid had a longer shelf-life than meat products containing high levels of linoleic acid.

### Sensory properties of frankfurters

**Figure 4:**
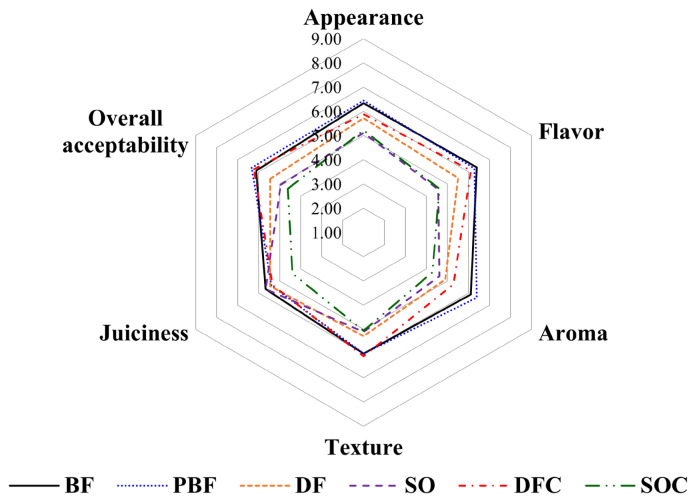
Sensory properties of frankfurters. The frankfurters were evaluated for their sensory properties as follows: appearance, flavor, aroma, juiciness, and overall acceptability using a 9-point hedonic scale (1 = extremely dislike and 9 = extremely like). BF (20% beef fat), PBF (20% pork back fat), DF (20% duck fat), SO (20% soybean oil), DFC (20% duck fat/1% κ-carrageenan), and SOC (20% soybean oil/1% κ-carrageenan).

Fat modification is a difficult technique for processed meat products because of the alterations in sensory properties. Hence, the sensory properties of lipid-modified meat products are normally tested to identify consumer acceptance [35]. In our study, sensory evaluation was performed using various frankfurter samples (Figure 4). The BF and PBF samples scored higher on their appearance compared to the other groups (p<0.05), primarily because of the differences in the instrumental color parameters of the samples. Instrumental color parameters have been known to be one of the most important factors when purchasing meat products [26]. For example, consumers tend to associate freshness with red color, whereas brown coloring is associated with stale meat [36]. The flavor, texture, and overall acceptability of the DFC samples were scored as those of the BF and PBF samples (p> 0.05). However, the DFC samples had lower aroma scores than the BF and PBF samples (p<0.05). This may potentially be attributed to the panelists’ unfamiliarity with the flavor of duck fat. Overall, the addition of duck fat and κ-carrageenan could be acceptable in flavor, texture, and overall acceptability of frankfurters.

## CONCLUSION

Our study demonstrate that duck fat and κ-carrageenan can be effectively used as beef fat and pork backfat substitutes in frankfurters. In comparison to the latter, duck fat contains higher levels of unsaturated fatty acids, along with lower levels of saturated fatty acids. Furthermore, the physicochemical properties of DFC frankfurters (emulsion stability, cooking loss, WHC, hardness, and rheological properties) were found to be quite satisfactory. Moreover, lipid oxidation was lower in duck fat-added frankfurters than in soybean oil-added frankfurters during storage at 4°C for 28 d. The organoleptic properties of DFC frankfurters were also found to be acceptable in flavor, texture, and overall acceptability. In summary, our data suggest that duck fat and κ-carrageenan can be employed as suitable options for producing meat products with modified or healthier fat.

## Figures and Tables

**Table 1:** Fatty acid profile of frankfurters and raw fat/oil

Fatty acid (%)	Frankfurter group^1)^						Raw fat/oil			
	
	BF	PBF	DF	SO	DFC	SOC	Beef fat	Pork back fat	Duck fat	Soybean oil
Caprylic (C8:0)	0.01±0.01	0.01±0.01	0.01±0.01	0.01±0.01	0.01±0.01	0.01±0.01	0.01±0.01	0.01±0.01	0.01±0.01	-
Capric (C10:0)	0.05±0.01^b^	0.07±0.01^a^	0.04±0.01^c^	0.02±0.01^d^	0.03±0.01^c^	0.02±0.01^d^	0.05±0.01	0.09±0.01	0.04±0.01	-
Lauric (C12:0)	0.11±0.01^b^	0.23±0.01^a^	0.11±0.01^b^	0.03±0.01^c^	0.10±0.01^b^	0.04±0.01^c^	0.12±0.01	0.30±0.01	0.12±0.01	-
Myristic (C14:0)	2.64±0.02^a^	1.63±0.02^b^	0.99±0.02^c^	0.26±0.01^d^	0.96±0.02^c^	0.26±0.01^d^	3.57±0.08^A^	1.87±0.02^B^	1.08±0.01^C^	0.09±0.01^D^
Pentadecylic (C15:0)	0.33±0.01^a^	0.10±0.01^b^	0.09±0.01^c^	0.02±0.01^d^	0.09±0.01^c^	0.02±0.01^d^	0.42±0.01^A^	0.11±0.01^B^	0.09±0.01^C^	0.02±0.01^D^
Palmitic (C16:0)	23.18±0.06^b^	22.87±0.01^c^	24.44±0.05^a^	12.56±0.02^d^	24.43±0.10^a^	12.49±0.07^d^	24.08±0.22^B^	22.64±0.34^C^	25.15±0.07^A^	11.12±0.02^D^
Heptadecanoic (C17:0)	0.68±0.01^a^	0.49±0.01^b^	0.18±0.01^c^	0.12±0.01^d^	0.19±0.01^c^	0.12±0.01^d^	0.72±0.01^A^	0.50±0.01^B^	0.17±0.00^C^	0.10±0.01^D^
Stearic (C18:0)	9.36±0.01^b^	11.26±0.05^a^	7.15±0.03^c^	5.25±0.05^d^	7.17±0.12^c^	5.19±0.05^d^	8.16±0.06^B^	10.88±0.71^A^	5.95±0.04^C^	4.43±0.01^D^
Arachidic (C20:0)	0.13±0.01^c^	0.19±0.01^b^	0.11±0.01^d^	0.35±0.01^a^	0.11±0.01^d^	0.35±0.01^a^	0.11±0.01^C^	0.19±0.01^B^	0.09±0.01^D^	0.35±0.01^A^
Heneicosylic (C21:0)	0.10±0.01^c^	0.50±0.01^a^	0.17±0.01^b^	0.10±0.01^c^	0.17±0.01^b^	0.10±0.01^c^	0.03±0.01^D^	0.47±0.01^A^	0.12±0.01^B^	0.05±0.01^C^
Saturated fatty acids	36.58±0.02^b^	37.34±0.02^a^	33.28±0.02^c^	18.72±0.02^d^	33.26±0.05^c^	18.60±0.03^d^	37.26±0.23^A^	37.06±0.23^A^	32.81±0.02^B^	16.15±0.01^C^
Myristoleic (C14:1)	1.38±0.07^a^	0.06±0.01^c^	0.12±0.01^b^	0.01±0.01^c^	0.12±0.01^b^	0.01±0.01^c^	2.36±0.06	0.07±0.01	0.18±0.01	-
Palmitoleic (C16:1)	5.20±0.02^a^	2.25±0.02^c^	3.12±0.03^b^	0.39±0.01^d^	3.10±0.05^b^	0.42±0.02^d^	5.94±0.02^A^	2.29±0.11^C^	3.52±0.03^B^	0.09±0.01^D^
Oleic (C18:1n-9, cis)	48.21±0.10^a^	41.06±0.08^c^	43.54±0.21^b^	24.01±0.19^d^	43.66±0.29^b^	23.90±0.15^d^	48.54±0.20^A^	40.77±0.40^C^	42.89±0.12^B^	21.77±0.01^D^
Elaidic (C18:1n-9, trans)	2.78±0.01^a^	2.70±0.01^b^	1.60±0.01^c^	1.55±0.04^d^	1.61±0.01^c^	1.61±0.03^c^	2.44±0.05^A^	2.52±0.08^A^	1.42±0.04^B^	1.47±0.01^B^
Linolelaidic (C18:2n-6, trans)	0.22±0.01^a^	0.05±0.01^b^	0.02±0.01^d^	0.01±0.01^e^	0.03±0.01^c^	0.01±0.01^e^	0.22±0.01	0.05±0.01	0.01±0.01	-
Linoleic (C18:2n-6, cis)	4.13±0.08^d^	14.11±0.08^c^	16.13±0.09^b^	49.25±0.20^a^	16.04±0.18^b^	49.30±0.27^a^	2.09±0.02^D^	15.05±0.45^C^	17.13±0.06^B^	54.24±0.05^A^
γ-Linolenic (C18:3n-6)	0.03±0.01^c^	0.05±0.01^b^	0.08±0.01^a^	0.02±0.01^d^	0.08±0.01^a^	0.05±0.01^b^	0.03±0.01^C^	0.04±0.01^B^	0.09±0.01^A^	0.02±0.01^C^
α-Linolenic (C18:3n-3)	0.17±0.01^d^	0.61±0.01^c^	0.84±0.01^b^	5.26±0.04^a^	0.84±0.01^b^	5.27±0.05^a^	0.11±0.01^D^	0.71±0.02^C^	0.98±0.01^B^	5.87±0.05^A^
c9, t11-CLA4) (C18:2)	0.30±0.01^a^	0.15±0.01^b^	0.06±0.01^c^	0.03±0.01^d^	0.06±0.01^c^	0.03±0.01^d^	0.37±0.01	0.18±0.01	0.07±0.01	-
Eicosenoic (C20:1)	0.45±0.01^b^	0.78±0.01^a^	0.43±0.03^b^	0.26±0.01^c^	0.43±0.02^b^	0.26±0.02^c^	0.37±0.02^B^	0.66±0.01^A^	0.34±0.03^B^	0.20±0.01^C^
Cis-8, 11, 14-Eicosadienoic (C20:3n6)	0.11±0.01^c^	0.12±0.01^b^	0.14±0.01^a^	0.05±0.01^d^	0.14±0.01^a^	0.05±0.01^d^	0.10±0.01	0.10±0.01	0.11±0.01	-
Arachidonic (C20:4n-6)	0.02±0.01^a^	0.02±0.01^a^	0.02±0.01^a^	0.01±0.01^b^	0.02±0.01^a^	0.01±0.01^b^	0.02±0.01	0.02±0.01	0.02±0.01	0.03±0.01
Eicosapentaenoic (C20:5n3)	0.31±0.01^c^	0.47±0.01^a^	0.44±0.01^b^	0.21±0.01^d^	0.45±0.02^b^	0.20±0.01^d^	0.11±0.01	0.29±0.02	0.31±0.01	-
Eicosadienoic (C20:2)	0.02±0.01^b^	0.09±0.01^a^	0.02±0.01^b^	0.01±0.01^c^	0.02±0.01^b^	0.02±0.01^b^	0.01±0.01	0.09±0.01	0.01±0.01	-
Eicosatrienoic (C20:3n-3)	0.01±0.01^d^	0.01±0.01^d^	0.02±0.01^c^	0.07±0.01^b^	0.02±0.01^c^	0.12±0.01^a^	0.01±0.01	0.01±0.01	0.02±0.01	-
Erucic (C22:1n-9)	0.02±0.01^c^	0.03±0.01^b^	0.04±0.01^a^	0.02±0.01^c^	0.04±0.01^a^	0.01±0.01^c^	0.02±0.01	0.03±0.01	0.05±0.01	-
Docosahexaenoic (C22:6n3)	0.05±0.01^d^	0.09±0.01^b^	0.08±0.01^c^	0.15±0.01^a^	0.07±0.01^c^	0.15±0.01^a^	0.03±0.01^C^	0.07±0.01^B^	0.07±0.01^B^	0.16±0.01^A^
Unsaturated fatty acid	63.42±0.03^d^	62.66±0.02^c^	66.72±0.05^b^	81.28±0.06^a^	66.74±0.08^b^	81.40±0.07^a^	62.74±0.05^C^	62.94±0.14^C^	67.19±0.03^B^	83.85±0.02^A^
Total	100.00	100.00	100.00	100.00	100.00	100.00	100.00	100.00	100.00	100.00

All values are represented as the mean±standard deviation of three replicates (n = 3).

CLA, conjugated linoleic acid.

1) BF, 20% beef fat; PBF, 20% pork back fat; DF, 20% duck fat; SO, 20% soybean oil; DFC, 20% duck fat/1% κ-carrageenan; SOC, 20% soybean oil/1% κ-carrageenan.

a–e Means within a row with different letters differ significantly from BF to SOC (frankfurter groups, p<0.05).

A–D Means within a row with different letters differ significantly from beef fat to soybean oil (raw materials, p<0.05).

**Table 2:** pH and color of frankfurters

Parameters		Frankfurter groups^1)^					

		BF	PBF	DF	SO	DFC	SOC
pH		6.07±0.01	6.07±0.01	6.06±0.01	6.06±0.01	6.06±0.01	6.06±0.01
CIE	L*	79.55±0.16^d^	80.78±0.33^c^	81.69±0.20^b^	82.23±0.47^a^	82.60±0.28^a^	82.52±0.40^a^
	a*	9.40±0.11^a^	8.75±0.14^b^	7.40±0.24^c^	6.66±0.13^e^	7.02±0.14^d^	6.79±0.10^e^
	b*	8.26±0.14^b^	7.75±0.15^c^	8.51±0.12^a^	8.27±0.24^b^	8.56±0.08^a^	8.53±0.19^a^

All values are represented as the mean±standard deviation of six replicates (n = 6).

CIE, Commission Internationale de l’Eclairage.

1) BF, 20% beef fat; PBF, 20% pork back fat; DF, 20% duck fat; SO, 20% soybean oil; DFC, 20% duck fat/1% κ-carrageenan; SOC, 20% soybean oil/1% κ-carrageenan.

a–e Means within a row with different letters differ significantly from BF to SOC (frankfurter groups, p<0.05).

**Table 3:** Emulsion stability and water holding capacity (WHC) of meat batters and cooking loss of frankfurters

Parameters		BF^1)^	PBF	DF	SO	DFC	SOC
Emulsion stability	Water released (%)	7.97±0.02^a^	5.58±0.35^bc^	5.05±0.29^c^	6.38±0.80^b^	2.93±0.57^d^	3.46±0.46^d^
	Fat released (%)	0.80±0.01	0.80±0.01	0.53±0.23	0.80±0.01	0.53±0.23	0.53±0.23
	Total released (%)	8.76±0.03^a^	6.38±0.35^bc^	5.58±0.51^c^	7.18±0.80^b^	3.46±0.80^d^	3.99±0.69^d^
WHC (%)		80.02±0.24^e^	82.11±0.45^d^	85.68±1.06^b^	83.9±0.89^c^	93.35±0.21^a^	92.95±0.42^a^
Cooking loss (%)		7.81±0.16^c^	7.90±0.42^c^	8.70±0.16^b^	9.21±0.41^a^	6.96±0.43^d^	7.49±0.24^c^

All values are represented as the mean±standard deviation of three replicates (n = 3).

1) BF, 20% beef fat; PBF, 20% pork back fat; DF, 20% duck fat; SO, 20% soybean oil; DFC, 20% duck fat/1% κ-carrageenan; SOC, 20% soybean oil/1% κ-carrageenan.

a–e Means within a row with different letters are significantly different (p<0.05).

**Table 4:** Texture profile analysis (TPA) of frankfurters

Parameters	BF^1)^	PBF	DF	SO	DFC	SOC
Hardness (g)	468.42±43.42^ab^	449.95±51.14^bc^	422.73±36.77^cd^	351.37±27.60^e^	499.88±16.63^a^	405.13±20.55^d^
Springiness	0.94±0.02^b^	0.98±0.01^a^	0.95±0.02^b^	0.93±0.02^b^	0.98±0.01^a^	0.98±0.01^a^
Cohesiveness	0.44±0.03	0.44±0.03	0.42±0.05	0.44±0.03	0.47±0.04	0.45±0.02
Chewiness (g)	187.40±11.95^bc^	193.75±26.63^ab^	165.79±12.34^cd^	144.27±16.41^d^	217.07±37.00^a^	180.99±14.34^bc^
Gumminess (g)	200.17±10.04^ab^	197.68±25.73^ab^	174.23±11.93^bc^	155.20±17.96^d^	221.40±37.52^a^	184.28±13.92^b^

All values are represented as the mean±standard deviation of six replicates (n = 6).

1) BF, 20% beef fat; PBF, 20% pork back fat; DF, 20% duck fat; SO, 20% soybean oil; DFC, 20% duck fat/1% κ-carrageenan; SOC, 20% soybean oil/1% κ-carrageenan.

a–e Means within a row with different letters are significantly different (p<0.05).
